# Increases in the longevity of desiccation-phase developing rice seeds: response to high-temperature drying depends on harvest moisture content

**DOI:** 10.1093/aob/mcv091

**Published:** 2015-07-01

**Authors:** K. J. Whitehouse, F. R. Hay, R. H. Ellis

**Affiliations:** ^1^T.T. Chang Genetic Resources Center, International Rice Research Institute, Los Baños, Philippines and; ^2^School of Agriculture, Policy and Development, University of Reading, Earley Gate, PO Box 237, Reading RG6 6AR, UK

**Keywords:** Seed longevity, genebank, rice, *Oryza sativa*, seed drying, seed development, desiccation phase, harvest moisture content.

## Abstract

**Background and Aims** Previous studies have suggested that the drying conditions routinely used by genebanks may not be optimal for subsequent seed longevity. The aim of this study was to compare the effect of hot-air drying and low-temperature drying on subsequent seed longevity for 20 diverse rice accessions and to consider how factors related to seed production history might influence the results.

**Methods** Seeds of rice, *Oryza sativa*, were produced according to normal regeneration procedures at IRRI. They were harvested at different times [harvest date and days after anthesis (DAA), once for each accession] and dried either in a drying room (DR; 15 % relative humidity, 15 °C) or in a flat-bed heated-air batch dryer (BD; 45 °C, 8 h d^–1^) for up to six daily cycles followed by drying in the DR. Relative longevity was assessed by storage at 10·9 % moisture content and 45 °C.

**Key Results** Initial drying in the BD resulted in significantly greater longevity compared with the DR for 14 accessions (seed lots): the period of time for viability to fall to 50 % for seeds dried in the BD as a percentage of that for seeds dried throughout in the DR varied between 1.3 and 372·2 % for these accessions. The seed lots that responded the most were those that were harvested earlier in the season and at higher moisture content. Drying in the BD did not reduce subsequent longevity compared with DR drying for any of the remaining accessions.

**Conclusions** Seeds harvested at a moisture content where, according to the moisture desorption isotherm, they could still be metabolically active (>16·2 %) may be in the first stage of the post-mass maturity, desiccation phase of seed development and thus able to increase longevity in response to hot-air drying. The genebank standards regarding seed drying for rice and, perhaps, for other tropical species should therefore be reconsidered.

## INTRODUCTION

Crop genetic resources comprising samples of landraces, modern and obsolete varieties, and their wild relatives are the biological basis of food security (FAO, 2013), and as such they are given high conservation priority (Maxted *et al*., 1997). Cultivated Asian rice (*Oryza sativa*) is the most important food crop of the developing world, being a staple for more than half the global population (FAO, 2003). Rice shows orthodox seed storage behaviour, meaning that the seeds can be dried and stored at low temperature and low moisture content in genebanks for long-term conservation (Ellis and Hong, 2007; Hay *et al*., 2013). At present there are >750 000 accessions of cultivated rice seeds (*O. sativa* and *Oryza glaberrima*) held in genebanks globally (FAO, 2010). The largest and most diverse collection (over 125 000 accessions) is stored in the International Rice Genebank (IRG) at the International Rice Research Institute (IRRI) in the Philippines. Although seeds remain viable for many decades under genebank storage conditions, over time their viability will decline and regeneration is required to maintain genetic integrity (Cromarty *et al*., 1982; Rao *et al*., 2006). It is therefore important that genebank staff regularly assess regeneration and management procedures to ensure that best practices are employed to conserve crop genetic resources for future generations.

The potential storage life of seeds is affected by pre- and post-harvest environments and practices (Hay *et al*., 2006; Probert *et al*., 2007). Seed quality traits (ability to germinate and survive air-dry storage) are acquired during the course of development and maturation (Hay and Smith, 2003; Probert *et al*., 2007). In many species, the ability of seeds to withstand desiccation to the low moisture levels required for storage occurs around mass maturity (end of seed-filling stage), but desiccation tolerance in rice is acquired earlier (Ellis and Hong, 1994). Although seeds can be stored as soon as they have become desiccation-tolerant, seed longevity in subsequent air-dry storage does not reach its maximum until some time later, during the desiccation phase of seed development (Ellis *et al*., 1993; Kameswara Rao and Jackson, 1996*a*, *b*; Hay and Smith, 2003; Ellis, 2011). During this desiccation phase, seeds become hygroscopic, i.e. their moisture status is now independent of the parent plant and instead is determined by ambient conditions (Ellis and Hong, 1994). Drying seeds at the end of this phase, once the seeds have equilibrated with ambient conditions (i.e. at harvest maturity), will reduce the rate of seed ageing thereafter and so maximize subsequent seed viability and longevity (Hay and Probert, 1995; Probert *et al*., 2007).

The 1994 Genebank Standards recommended that seeds should be stored hermetically at a moisture content (m.c.) of 3–7 % of fresh weight (depending on seed oil content) and −20 °C (FAO/IPGRI, 1994). In these conditions the rate of ageing is slow and viability can be maintained for long periods (Ellis *et al*., 1989, 1992; Ellis and Hong, 2006; Gómez-Campo, 2006; Pérez-García *et al*., 2009; Hay *et al*., 2013). To achieve this low moisture content, it was further recommended that seeds of orthodox species should be dried immediately after harvest in a drying chamber at 10–15 % relative humidity (RH) and 10–25 °C (FAO/IPGRI, 1994). More recently, this was modified to 10–25 % RH and 5–20 °C (FAO, 2013). A relatively low drying temperature was adopted to reduce the rate of ageing during the drying process, particularly when seeds still have high m.c. and/or for species with seeds easily damaged by high-temperature drying (Cromarty *et al*., 1982). However, it has been suggested that, in particular for tropical species, a low drying temperature may curtail late developmental processes in seeds and have a negative impact on subsequent longevity in storage (Hay, 1997). A preliminary study showed that initial intermittent high temperature drying (45–50 °C), before drying at 15 % RH, 15 °C, resulted in greater subsequent seed quality than drying throughout at 15 % RH, 15 °C in rice (Crisostomo *et al*., 2011). The aim of this study was to evaluate the effects of initial high-temperature drying of rice seeds from 20 diverse varieties for different periods before subsequent drying to equilibrium in the genebank dry room, compared with drying solely in the dry room, on subsequent longevity in storage. We further considered why the response varied between the different varieties (seed lots).

## MATERIALS AND METHODS

### Plant material

Seeds of 20 rice (*Oryza sativa*) accessions representing five variety groups (aus, aromatic, indica, and temperate and tropical japonica; McNally *et al*., 2009) (Table 1) were sampled from the IRG active collection and held at 50 °C for 5 d to break dormancy. They were sown on 23 November 2012 and transplanted on 18 December 2012 into plots on the IRRI experimental station. Normal rice production practices and routine plant protection measures were followed. Seed lots were harvested between March and April 2013. The target harvest time was 35 d after 50 % anthesis (DAA). However, due to biological/environmental (e.g. early shattering, late tillering) and/or workload constraints the actual harvest times were between 24 and 48 DAA (Table 1).

**Table 1.** Information on the 20 rice seed lots used in the study showing date of harvest, the interval from 50 % anthesis to harvest date (DAA), initial seed moisture content (m.c.) and equilibrium relative humidity (eRH) at harvest

Accession	Variety name	Variety group^1^	Harvest date	DAA^¶^	Moisture content (s.e.), % fresh weight	eRH, %
IRGC 117264	Azucena	Tropical japonica	19 March	24	22·4 (0·42)	95·9
IRGC 117265	Dom-sufid	Aromatic	11 March	24	22·7 (0·09)	96·1
IRGC 117266	Dular	Aus	19 March	37	18·9 (0·11)	92·9
IRGC 117267	FR 13 A	Aus	4 April	36	16·8 (0·22)	88·4
IRGC 117268	IR64-21	Indica	2 April	44	14·9 (0·04)	74·4
IRGC 117269	Li-Jiang-Xin-Tuan-Hei-Gu	Temperate japonica	11 March	38	26·8 (0·36)	96·9
IRGC 117270	M 202	Temperate japonica	14 March	38	23·4 (0·23)	97·4
IRGC 117271	Minghui 63	Indica	15 April	33	16·7 (0·06)	91·6
IRGC 117272	Moroberekan	Tropical japonica	10 April	35	17·7 (0·05)	91·6
IRGC 117273	N 22	Aus	5 March	29	20·8 (0·12)	91·9
IRGC 117274	Nipponbare	Temperate japonica	5 March	40	28·9 (0·31)	96·0
IRGC 227275	Pokkali	Indica	27 March	37	13·7 (0·02)	69·8
IRGC 117276	Sadu-cho	Indica	27 March	26	13·2 (0·09)	67·8
IRGC 117277	Sanhuangzhan no. 2	Indica	10 April	38	16·2 (0·04)	86·5
IRGC 117278	Swarna	Indica	4 April	36	18·2 (0·28)	91·7
IRGC 117279	Tainung 67	Temperate japonica	15 April	45	17·3 (0·08)	86·7
IRGC 117280	Zhenshan 97B	Indica	14 March	38	23·3 (0·24)	96·1
IRGC 117281	Aswina	Indica	25 March	48	19·3 (0·14)	94·6
IRGC 117282	Cypress	Tropical japonica	25 March	41	18·8 (0·04)	92·8
IRGC 117283	Rayada	Aus	2 April	34	16·5 (0·16)	83·8

^1^Variety group taken from McNally *et al*. (2009).

^¶^Estimated from time of mid-flowering to harvest date.

Immediately after harvest, the seeds were threshed and blown to remove debris. A sample taken at random from each accession was placed inside a 3·2-mL sample holder in the measuring chamber of an AW-D10 water activity station used in conjunction with a HygroLab 3 display unit (Rotronic South East Asia, Singapore). The temperature and equilibrium relative humidity (eRH) were measured once the reading had stabilized, after 20–40 min. Seed m.c. (fresh weight basis) was determined using three 5-g samples from each accession using the high-constant-temperature oven method (ISTA, 2013). The samples were ground in a Krups 75 coffee grinder and weighed before being placed at 130 °C for 2 h. The samples were removed from the oven and placed over silica gel for 1 h to cool before reweighing.

### Seed drying

Seeds from each accession were divided into a maximum of seven 300-g samples (depending on the quantity available) and placed into 0·2 × 0·33 m (length × width) nylon mesh bags (1-mm diameter holes). They were stored inside sealed 0·6 × 0·3 × 0·132 m (length × width × height) electrical enclosure boxes (Ensto Finland Oy) at room temperature (∼22·5 °C) overnight. The following morning (0800 h), one sample was immediately placed in the genebank dry room (DR; 15 % RH, 15 °C) and the remaining samples (up to six) were placed into a locally fabricated flat-bed batch dryer (BD) at the IRRI experimental station. The change in weight and eRH of the DR samples was monitored daily. In the BD, air was heated (to ∼45 °C) with a burner fuelled by kerosene and blown into a chamber below the seeds before being driven up through the perforated base on which the seeds were placed. Seeds were exposed to 8 h of heated-air drying (0800–1600 h) per daily (24-h) cycle. At the end of this 8-h period, one sample was removed and a small subsample (∼15 g) was taken to determine seed eRH and m.c., as before. The remainder of the seeds of this sample was transferred within the nylon mesh bag to the DR, where all seed samples completed drying (i.e. equilibrating to 15 % RH, 15 °C; resulting in an m.c. of 6–7 %). The remaining 300-g samples were sealed inside 0·6 × 0·3 × 0·132 -m (length × width × height) electrical enclosure boxes at room temperature overnight (1600–0800 h) before they were returned to the BD for the next 8-h heated-air treatment period. Each accession consisted of different seed samples that had been dried using the BD for up to six daily cycles. This protocol resulted in all samples being dried to the same m.c. but individually differing in the number of daily heated-air drying cycles in the BD (0–6 d). Once equilibrated in the DR (up to 14 d), samples were sealed inside 0·17 × 0·12 -m (length × width) laminated aluminium foil packets and stored at 2–4 °C until experimental seed storage began in June 2013.

### Seed storage

Seeds of each treatment combination [accession (20) × drying treatment (7)] were removed from cold storage (2–4 °C) and equilibrated to room temperature (22·5 °C) before opening. Each sample was split into 5-g subsamples (maximum of 29) and placed into 30-mm-diameter open Petri dishes and held over a non-saturated LiCl solution (60 % RH) in a sealed 0·6 × 0·3 × 0·132-m (length × width × height) electrical enclosure box for 7 d at 22·5 °C. The RH provided by the solution was checked at weekly intervals, using the water activity-measuring instrument described above, and the bulk solution was adjusted if necessary by adding distilled water, stirring and allowing equilibration before re-checking RH (Hay *et al*., 2008).

Seed m.c. reached equilibrium with this environment after 7 d. Four 5-g subsamples from each treatment combination were taken and seed eRH was measured. Three of these subsamples were used to determine m.c. and the fourth to estimate initial ability to germinate (prior to experimental storage). The remaining 5-g subsamples were each sealed inside 0·12 × 0·09-m (length × width) laminated aluminium foil packets and then placed in an incubator at 45 °C. One packet per treatment combination was removed at 1- to 3-d intervals up to 45 d for germination testing (see below). For some seed lots, in which viability was lost before 45 d, sampling was discontinued earlier; for a few seed lots, later samples were at longer intervals due to an unexpectedly slow rate of viability loss. At 21 d (mid-storage) and at the end of the storage experiment, m.c. was determined using three additional 5-g packets of seeds each time.

### Seed germination

Ability to germinate was estimated with four replicates of 30 seeds, sown on two layers of Whatman No. 1 paper wetted with 7·5 mL distilled water in 90-mm-diameter Petri dishes. They were incubated at constant 30 °C (12 h light and 12 h dark cycle). Germination was scored after 2, 3, 4, 5, 7 and 14 d. Non-germinated seeds were dehulled and tested for an additional 7 d before final scoring. Seeds were scored as germinated when the radicle had emerged by at least 2 mm.

### Statistical analyses

Seed survival (ability to germinate after different periods of air-dry storage in the experimental regime) curves were fitted by probit analysis using GenStat for Windows, Version 15 (VSN International Ltd., Oxford, UK), thereby fitting the following equation to estimate the period (days) for viability to fall to 50 % (*p*_50_), *K*_i_ and *σ*:

(1) $$v=K_{\text{i}}–\;\left(p/\sigma \right)$$

where *v* is the viability (ability to germinate) in normal equivalent deviates (NED) of a seed lot stored for a period of *p* (days), *K*_i_ is the initial viability (NED) and σ (days) is the standard deviation of the normal distribution of seed deaths in time (Ellis and Roberts, 1980). The estimate of *p*_50_ was used as the measure of longevity. For those accessions also showing loss in dormancy during (early) storage, i.e. after-ripening, a probit model combining loss in dormancy with loss in viability was applied:

(2) $$g=\;(K_{\text{d}}+\beta_{\text{1}}p)\;\times \;\left[K_{\text{i}}–\;\left(p/\sigma \right)\right]$$

where *g* is ability to germinate (NED), *p*, *K*_i_ and *σ* are as in eqn (1), *K*_d_ is the initial proportion of non-dormant seeds (NED) and *β*_1_ is the probit rate of loss of dormancy (Kebreab and Murdoch, 1999). Eqn (2) was fitted using the FITNONLINEAR directive in GenStat. Probit analysis was carried out for all seed lots within an accession simultaneously, fitting the full model (different estimates for all parameters) and reduced models in which one or more parameters were constrained to a common value for all seed lots. An approximate *F*-test was used to determine the best model.

The difference in longevity (*p*_50_) between the highest value from the BD treatments (BD *p*_50_) and the DR treatment (DR *p*_50_) was calculated as a proportion of the DR *p*_50_ according to the equation: 100 × ((BD *p*_50_ – DR *p*_50_)/DR *p*_50_). Split-line regression analysis was used to explore the relationships between different variables and relative difference in longevity. A modified version of the D’Arcy-Watt equation (D’Arcy and Watt, 1970) was used to describe the relationship between seed m.c. [converted to water content (WC) as a proportion of dry weight] and eRH, as follows (also fitted using the FITNONLINEAR directive in GenStat):

(3) $$\text{WC}=y+c\rho /\rho_{o}+\frac{k'k\left(\text{eRH}/100\right)}{1+k\left(\text{eRH}/100\right)}$$

where *c*, *k* and *k′* are parameters that relate to the number and strength of weak (*c*) and multi-molecular (*k*, *k*′) water-binding sites. Since there were few data at very low water contents, the part of the original equation relating to strong water binding sites was replaced with *y*:

(4) $$y=\frac{K'K\left(\text{eRH}/100\right)}{1+K\left(\text{eRH}/100\right)}$$

The fitted equations were transformed to a fresh weight basis for plotting.

### Additional data

Data from another, independent experiment carried out on seeds produced from plants of the same accessions and cultivated at the same time and close to the plants referred to above, are also presented in this paper. The original data are reported elsewhere (Hay *et al*., 2015). Briefly, all the methods, including the seed storage protocol and analysis, are as described here with the exception that seeds were harvested at 24, 31, 38 and 45 DAA and all seed lots were immediately dried in the DR.

## RESULTS

### Seed drying

**Fig. 1.**
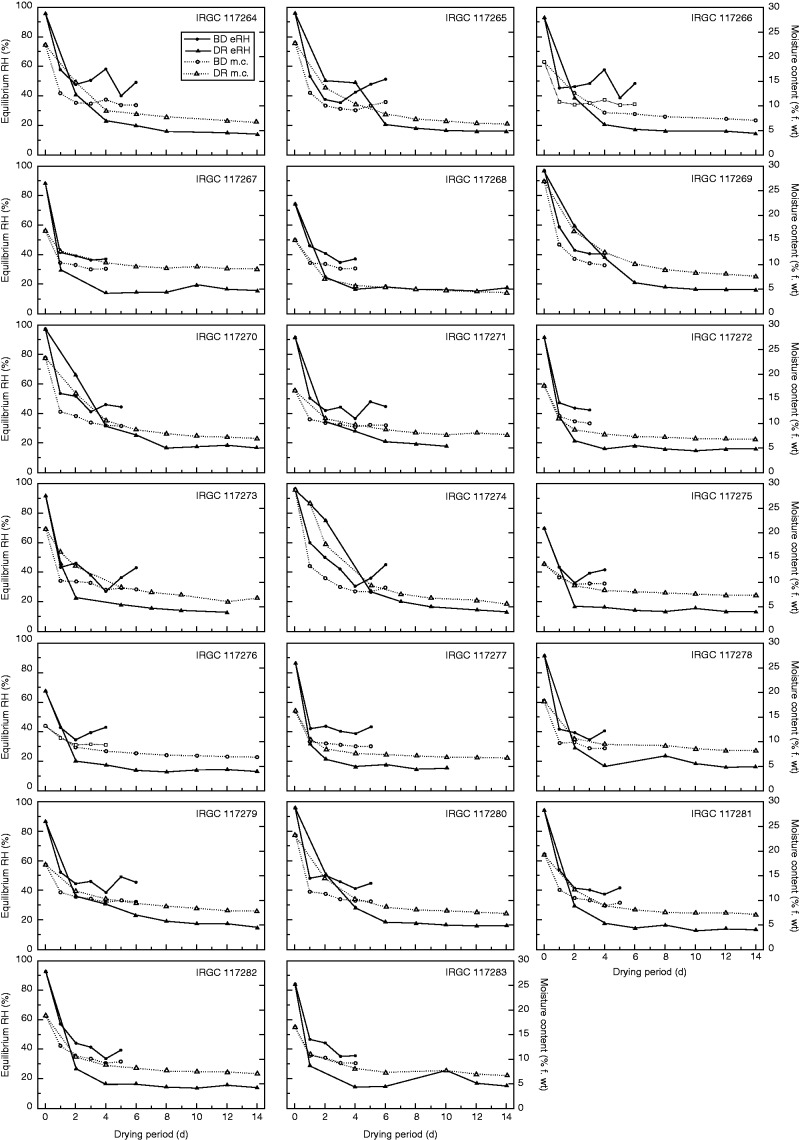
Drying curves for 20 rice accessions. Initial moisture content (m.c., %, fresh weight) and equilibrium relative humidity were measured before freshly harvested seeds were placed either in the dry room (DR) or the flat-bed dryer (BD; 8 h d^–1^). Moisture content for the BD seeds was determined using the high-temperature oven method (ISTA, 2013). For seeds dried in the DR, m.c. was estimated based on the initial moisture content and change in sample weight.

The pattern of loss in moisture for both drying regimes and for all seed lots showed the expected trend of a negative exponential followed by approximate equilibrium (Fig. 1). Seeds immediately placed in the DR did not dry as rapidly over the first day as those initially placed in the BD, with the exception of accessions IRGC 117268, −72, −75, −76, −77 and −83, for which drying rates were similar for BD and DR samples. Seeds dried in the BD had a mean eRH of 49·3 % (s.e. 1·4) and m.c. of 11·4 % (s.e. 0·3) after the first 8-h period, while seeds dried in the DR had a mean eRH of 52·1 % (s.e. 12·6) and m.c. of 14·6 % (s.e. 2·5) after 1 d. Seeds dried in the DR varied considerably in eRH (17·3–7·5 %) after the first 2 d. The period for seeds to reach equilibrium in the DR [15 % RH, 15 °C; 6·1 % m.c. as estimated in the Seed Viability module of the Seed Information Database (Royal Botanic Gardens Kew, 2008)] ranged from 4 to 14 d, whereas this was 4–5 d for seeds in the BD (∼40 % eRH, 8·4 % m.c.). Thereafter, m.c. fluctuated, with the regaining of moisture observed in some cases.

### Seed moisture isotherm

**Fig. 2.**
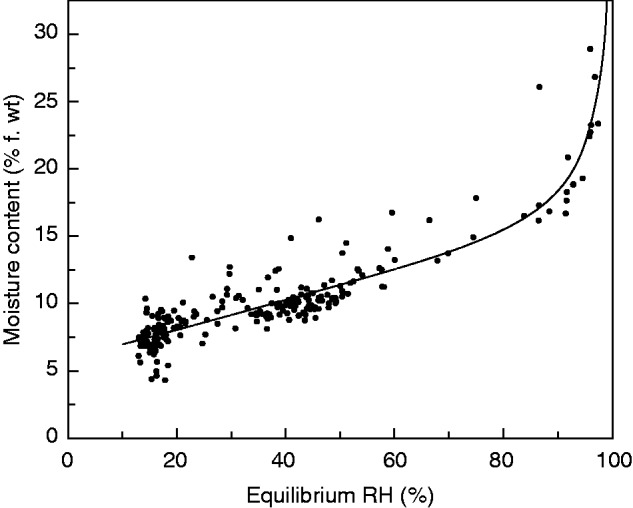
Relationship between seed moisture content and equilibrium relative humidity (eRH) during seed drying for 20 rice accessions (data shown in Fig. 1). All eRH measurements were made at between 20·8 and 24·7 °C. Seeds were dried either immediately in the dry room or initially dried in the batch dryer. The solid line is the result of fitting a modified version of the D’Arcy-Watt isotherm equation (eqn 3).

The desorption isotherm for all seed lots showed a shallow slope between 13 and 80–85 % eRH (7·3 and 15.5–16·6 % m.c.; Fig. 2). The m.c. then increased rapidly with further increase in eRH.

### Seed longevity

The mean seed moisture content during experimental storage across all seed lots (accession × drying treatment) was 10·9 % (s.e. 0·1).

Seeds of some accessions showed dormancy that was lost during early experimental storage (accessions IRGC 117264, −65, −66, −67, −73, −75, −81 and −83); all seed lots ultimately showed a loss in viability (Supplementary Data Fig. S1). These changes in ability to germinate during storage were quantified by either eqn (1) or (2) (Appendix).

Differences in seed longevity were apparent between accessions and, in some cases, amongst the different drying treatments within accessions (Appendix; Supplementary Data Fig. S1). Three categories of within-accession variation were apparent. For accessions IRGC 117268, −71, −72, −75, −77 and −83, there were no differences in *K*_i_ or σ amongst any of the seven different drying treatments. For accessions IRGC 117264, −65, −66, −69, −70, −74, −79 and −82, there were significant differences in *K*_i_ and σ (*P*  <  0.05) between BD and DR treatments, but not amongst different BD treatments (i.e. initial period of BD drying). For the six remaining accessions (IRGC 117267, −73, −76, −78, −80 and −81), it was not possible to constrain *K*_i_ and σ to common values for seeds given different drying treatments.

Where it was not possible to constrain *K*_i_ and σ to common values for BD and DR treatments, at least one of the BD treatments (period of drying in the BD) resulted in an improvement in longevity (*p*_50_) compared with drying in the DR (Appendix). For example, for accession IRGC 117267 the estimate of *p*_50_ was 63·7 d for seeds first dried for 3 d in the BD and 48·7 d for seeds dried throughout in the DR, and for IRGC 117264 the estimate of *p*_50_ was 29·6 d for seeds first dried in the BD and 14·6 d for seeds dried throughout in the DR. For the accessions where *K*_i_ and σ could not be constrained across BD treatments, most accessions showed an improvement in *p*_50_ after the first day in the BD compared with the DR, which was either then maintained or increased (Appendix) until the day the seeds reached equilibrium in the BD (Fig. 1). For example, for accession IRGC 117281 the estimate of *p*_50_ was 33·1 d after the first 8 h in the BD and by the end of the fourth daily cycle it had increased to 42·4 d, when seeds had reached the minimum eRH of 37·4 %.

**Fig. 3.**
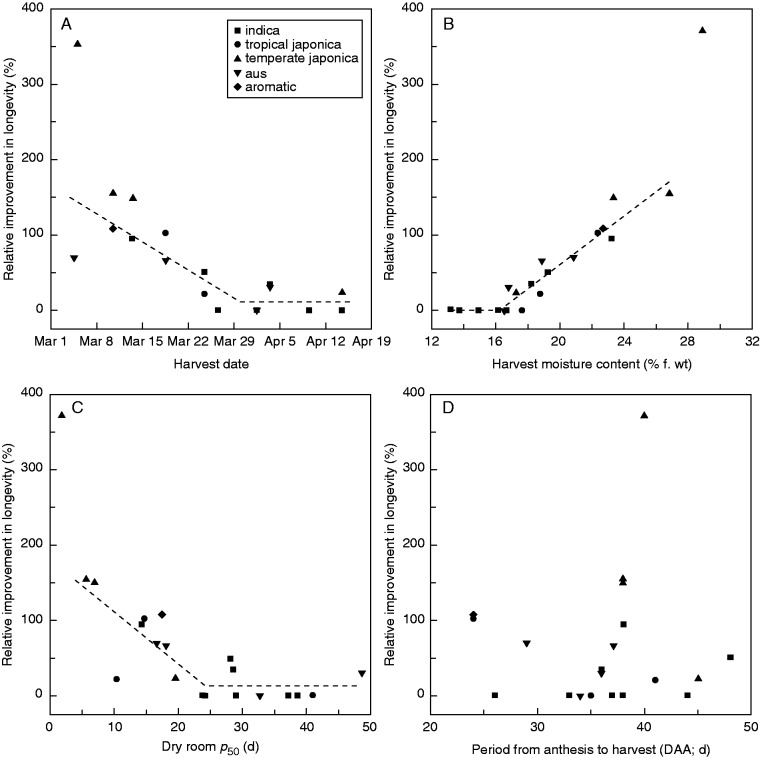
Split-line relationships between the relative improvement in longevity (%) between the two drying treatments [flat-bed dryer (BD) *p*_50_/dry room (DR) *p*_50_] for 20 rice accessions and (A) harvest date, (B) seed harvest moisture content and (C) DR *p*_50_. The outlying data point (372 % relative improvement in longevity;  accession IRGC 117274) was not included in the analyses. No relationship was apparent between improvement in longevity and period from anthesis to harvest (D). A relative improvement in longevity of 100 % is equivalent to a doubling in longevity of BD compared with DR treatments.

The improvement in longevity of seeds initially placed in the BD relative to those dried in the DR throughout ranged from 0 % (accessions IRGC 117268, −71, −72, −75, −77 and −83) to 372 % (accession IRGC 117274) (Appendix; Fig. 3). The improvement was more than 100 % (i.e. longevity was more than doubled) for five of the 20 accessions. These highly variable differences in subsequent seed longevity depending on drying treatment amongst the 20 accessions were further examined by investigating the possibility that they might be dependent upon known differences in their seed production history. Split-line regressions accounted for 66·3, 85 and 65·8 % of the variance in the case of the relationship between improvement in longevity and harvest date, harvest moisture content and DR *p*_50_, respectively (Fig. 3A–C). The respective breakpoints occurred on 30 March 2013, at a harvest m.c. of 16·2 % or a DR *p*_50_ of 24·2 d. There was no relationship apparent between the improvement in longevity and DAA (Fig. 3D).

## DISCUSSION

Recommendations for the management of genebank accessions emphasize the importance of initial seed drying to extend the subsequent longevity of seeds during storage (Cromarty *et al*., 1982; FAO/IPGRI, 1994; Rao *et al*., 2006; FAO, 2013), but as far as we are aware there has not been a critical evaluation (impact on subsequent quality or longevity of the seeds) of the conditions that are actually used by genebanks for any particular species. Rather, the recommendation to dry seeds in genebanks to low (∼5 %) m.c. using cool temperatures combined with very low RH (Cromarty *et al*., 1982) was driven by the requirement for a single, simple, safe procedure for diverse species in all locations worldwide. It was determined by combining the seed viability equation, developed and quantified from investigations with mature seeds, with equations describing the effect of environment on seed drying rate and seed temperature in constant-temperature heated-air dryers in contrasting species. In this context we note that, for example, seeds of onion (*Allium cepa*) are vulnerable to high seed-drying temperature: according to North (1948), air temperature should not exceed 32 °C at 12–20 % moisture content or 21 °C if moisture content is >20 %.

The current study used alternate temperature cycling (8 h high/16 h low) for the high-temperature treatment and it is clear that, in the case of rice, subsequent longevity might be improved >3-fold if seeds are not initially dried in the conditions used by the genebank at IRRI, which reflect genebank standards at the time of installation (Appendix; Fig. 3; Supplementary Data Fig. S1). This is in agreement with the preliminary study by Crisostomo *et al*. (2011) of a similar comparison. Such improvements in rice seed longevity could potentially greatly reduce the number of genebank accessions that have to be regenerated each year due to declining viability.

In general, seeds dried in the BD initially dried more quickly, but did not reach m.c. as low as those obtained when seeds were dried in the DR, with increases in seed moisture observed after 3–4 d (Fig. 1). The BD lacks a dehumification system and is operated in an open environment. Since the ambient conditions in the dry season at IRRI are warm (25–30 °C) and humid (80–90 %), even when the air is heated there is a limit to the extent to which the RH can be reduced, and hence how effective it will be for drying seeds to low moisture contents. It is therefore perhaps surprising that for 14 of the 20 accessions there was such a benefit of drying with the BD compared with the DR and, furthermore, that the benefit was maintained over several drying cycles (Appendix; Supplementary Data Fig. S1), even after there were increases in seed m.c. in the equilibrium phase (Fig. 1).

Seeds of the different accessions did not respond in the same way (Appendix; Supplementary Data Fig. S1). This was not obviously related to variety group, although the improvement in longevity when seeds were dried in the BD compared with the DR was greatest for three of the four temperate japonica varieties (Fig. 3). The seeds of these accessions had the lowest *p*_50_ when they were dried throughout in the DR (Fig. 3C). Seeds of temperate japonica varieties are known to be short-lived in storage (Ellis *et al*., 1992, 1993; Kameswara Rao and Jackson, 1997; Xue *et al*., 2008; Hay *et al*., 2013), but it seems that it might be possible to improve the longevity of seeds of such accessions more than those of non-temperate japonica varieties by changing the drying regime. That is, one reason why they are so short-lived, especially when regenerated in a tropical environment, may be because genebank drying conditions are not optimal for drying seeds of these varieties in particular. In terms of current practice, we suggest that genebanks using low-temperature, low-humidity environments to dry rice seeds delay harvesting accessions until after m.c. has declined naturally to below ∼16 %, if ambient conditions allow, since at and below this value the contrasting drying temperatures provided similar longevity (Fig. 3B). If seeds are unlikely to dry to this m.c. due to high ambient humidity, high-temperature drying might be superior with respect to seed longevity.

**Fig. 4.**
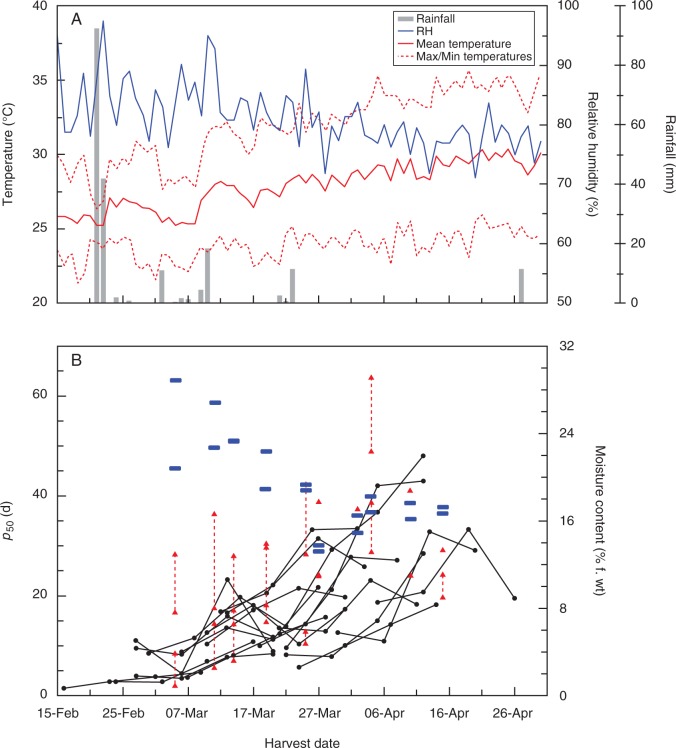
(A) Climate data for the IRRI experimental station over the harvesting period (provided by the IRRI Climate Unit; see key). (B) Changes in the period for viability to fall to 50 % (*p*_50_) for seeds harvested on different days at 24, 31, 38 and 45 DAA and dried in the dry room (black circles; data from Hay *et al*., 2015) and for seeds from the main experiment described in this paper, harvested on the date indicated at a similar DAA (±0–3 d) and dried in the dry room throughout (lower red triangle) or in the flat-bed dryer (BD, 8 h d^–1^; upper red triangle; result shown is for the optimum number of drying days). If there is no connecting dashed line, there was not a significant improvement in longevity with the BD treatment. The harvest moisture contents of seeds of the 20 accessions in this investigation are also shown (short blue horizontal lines).

This is not the first time that an alternative drying regime has been reported to be better than standard genebank DR conditions (15 % RH, 15 °C) for subsequent seed longevity or quality. As well as the previous research on rice by Crisostomo *et al*. (2011), Butler *et al*. (2009) described how the longevity of seeds of foxglove (*Digitalis purpurea*) that were intentionally harvested prematurely, in the post-abscission (i.e. desiccation) phase of seed development, increased when seeds were dried at RH >15 %. It has been well reported how seed quality, including longevity, increases during the desiccation phase of seed development, and results from gene expression and metabolite studies have shown that seeds are metabolically active during this phase (Angelovici *et al*., 2010 and references therein). Chatelain *et al*. (2012) further suggested, based on proteomic studies, that the desiccation phase from the end of seed-filling (mass maturity) onwards should be divided into two, the first when there is increasing seed longevity and then a final, maturation drying stage. Most studies of seed development involve the harvesting of seeds from a single plant population, be that a wild population or one that is cultivated for such experiments. Coincident to the primary experiments reported here, additional plants of the same accessions were cultivated in adjacent plots. These plants flowered within a day or so of the plants that are the main subject of this paper, but were harvested on different occasions during seed development (24, 31, 38 and 45 DAA). Based on data from Kameswara Rao and Jackson (1996*a*, *b*), we can assume that these seeds (like those of the main study) had reached mass maturity and were therefore in the desiccation phase of seed development. Similar to these and other studies, within each accession, there were increases in *p*_50_ the later the seeds were harvested, with, in some instances, a decrease in longevity at the final harvest (indicative of on-plant ageing; Fig. 4). However, across the 20 seed lots produced for the main study, there was not a significant relationship between DR *p*_50_ and DAA (graph not shown), nor between relative improvement in longevity with the BD treatment and DAA (Fig. 3D). This suggests that DAA alone does not determine seed longevity. Plotting the *p*_50_ data from the two experiments against harvest date, it is clear that there is a general trend of increase in *p*_50_ following drying in the DR and harvest date over the total harvest period, sometimes with that decline in longevity for particular accessions at the last harvest (Fig. 4). It also appears that it was only seeds that were harvested earlier in the season (i.e. in March) that responded positively to the BD treatment (Fig. 3A). These seeds were also the seeds that happened to have the lowest *p*_50_ for DR-dried seeds and, perhaps most importantly, an m.c. >16.2 % (Figs 2B and 4). This coincides with the part of the moisture desorption isotherm above which, seeds become metabolically active, i.e. at eRH >80–85 % (Fig. 2) (Vertucci and Leopold, 1984; Walters *et al.*, 2002).

Taking the results from the two experiments together, it appears that seeds harvested later in the season and irrespective of DAA had already acquired greater longevity due to on-plant drying, i.e. they had already entered the first, ‘increasing longevity’ part of the desiccation phase (Chatelain *et al*., 2012), triggered by decline in ambient RH and a long dry phase (without rain) after the end of March (coinciding with the breakpoint shown in Fig. 2A). If the seeds had already dried on the plant to an m.c. at which they were no longer metabolically active, drying at a higher temperature (i.e. in the BD) did not improve the longevity compared with seeds directly dried directly in the DR, but nor did it have a negative effect. We suggest that rice seeds do not strictly follow a sequence of development with respect to time (DAA) once they have reached mass maturity; rather, due to the high humidity of the growing environment at IRRI, the rice seeds stay in a pre-desiccation state in which increases in longevity are limited. It is only when they experience some desiccation that substantial ‘accumulation of seed longevity’ is activated. If rice seeds regenerated at IRRI for long-term storage in the genebank are harvested in the dry season before ambient RH has decreased and hence with high m.c. (>16.2 %), they should be dried at a high temperature to allow continued metabolism, such as the accumulation of proteins that may be involved in stabilizing tissues during seed storage. If they have already dried to m.c. <16·2 %, they are perhaps in that second, final maturation drying phase (Chatelain *et al*., 2012) and will not respond to high-temperature drying. These results also, not for the first time, raise important questions about the value of using single seed lots to make comparisons of seed longevity between genotypes (e.g. for genetic association studies), even if they are harvested at the same ‘stage’ (DAA) of seed development.

Very moist seeds are expected to be more sensitive to damage in heated-air dryers than seeds with low m.c. (Nellist, 1980; McDonald and Copeland, 1997). This represents an apparent contradiction to the results presented here. However, evaporative cooling by the moist seeds may have maintained seed temperatures very much cooler than the 45 °C air temperature, and closer to the ambient temperature (∼30 °C; Fig. 4), when seeds would normally dry *in situ*. In contrast, the genebank drying room temperature of 15 °C is very much cooler than the seeds would experience *in situ*, and again the actual temperature experienced by the seeds might be reduced further due to evaporative cooling. Moreover, traditional heated-air drying investigations used mature seeds with moist samples created by ‘wetting up’ dry seeds, or perhaps from harvests delayed by heavy rain well beyond harvest maturity. Such differences could well explain the apparent contradictions with the conventions of heated-air commercial seed drying. The results also appear to contradict the damaging effect of high seed production temperatures on developing and maturing seeds of rice (Ellis *et al*., 1993; Kameswara Rao and Jackson, 1996*a*). However, Kameswara Rao and Jackson (1996*a*) did suggest that planting should be timed so that seeds ripen when weather conditions are both cool and dry, based on the rainfall data for that year (1994 dry season harvest). Indeed, in that study there was significant rainfall by the time the last harvest was made, in May, although it should be noted that ambient RH that year (not presented in that publication but obtained from the IRRI Climate Unit) did not reach the same low values as in March–April 2013. Furthermore, recent investigations point to the phase of rice seed quality development most sensitive to high temperature being before the end of the seed-filling phase (Ellis, 2011) and possibly as early as the histodifferentiation phase soon after pollination (Martínez-Eixarch and Ellis, 2014). It should also be noted that, in addition to the higher temperature, seeds in the BD would also have been exposed to higher RH than the 15 % of the DR. It has been reported elsewhere, most notably for pre-dispersal seeds of foxglove, that drying at 15 % RH is not optimum for subsequent seed storage longevity (Hay and Probert, 1995; Butler *et al*., 2009); thus, both RH and temperature during drying may be important for the accumulation of seed longevity. Determining an optimum drying regime and/or potentially customizing drying regimes depending on production history (e.g. DAA, harvest m.c.) might also be difficult since these variables are not independent: the rate of drying will depend on both the temperature and the RH of the air (and indeed the flow of air around the seeds), and changing the temperature of the air will also change the RH. It should not be forgotten, however, that even if a different initial drying regime is identified as being better that the current regime, it may still be necessary to equilibrate the seeds after the initial drying, to ensure the seeds are at an appropriate m.c. for long-term storage.

To conclude, there is clear evidence that, for rice, initial drying with hot air, for example by using a flat-bed batch dryer, can result in seed lots with significantly greater subsequent longevity in storage than for those dried immediately under low-temperature, low-humidity conditions, particularly if the seeds are harvested at a time when their m.c. is relatively high. If this is confirmed in independent studies, which we suggest are now required, it may prove necessary to amend genebank standards for seed drying in order to ensure seeds have maximum longevity when they are first placed into storage.

## SUPPLEMENTARY DATA

Supplementary data are available online at www.aob.oxfordjournals.org and consist of Figure S1: percentage germination for seeds dried immediately in the dry room or initially dried in the batch dryer together with lines fitted according to eqns 1 and 2.

Supplementary Data

## APPENDIX

Results of fitting models to quantify changes in ability to germinate during hermetic storage at 45 °C and m.c. shown for 20 *O. sativa* accessions. Eqn (1) is the viability equation; eqn (2) is for combined loss in dormancy and loss in viability (Supplementary Fig. S1). Samples were immediately dried in the DR or initially in the BD for 1 (BD1), 2 (BD2), 3 (BD3), 4 (BD4), 5 (BD5) or 6 (BD6) d. The parameters shown are for the simplest model (fewest parameters) that could be fitted without a significant (*P* = 0·05) increase in residual deviance compared with the best-fit model. The m.c. is the mean and s.e. calculated from measurements taken at three stages across the duration of the storage experiment.

**Table:**

			Loss in dormancy		Loss in viability			
Treatment	Model	Seed m.c. (s.e.), % fresh weight	*K*_d_ (s.e.), NED	β_1_ (s.e.) (d)	*K*_i_ (s.e.), NED	σ^–1^ (s.e.), d^–1^	*p*_50_, d	Difference in *p*_50_ relative to DR, %, d
IRGC 117264								
BD1	Eqn (2), *K*_d_, β_1,_ *K*_i_ and σ^–1^ constrained within BD treatments	10·8 (0·04)	0·81 (0·62)	0·29 (1·37)	4·61 (0·72)	0·16 (0·04)	29·6	102·7
BD2		10·8 (0·03)						
BD3		10·9 (0·03)						
BD4		10·8 (0·03)						
BD5		10·8 (0·02)						
BD6		10·8 (0·03)						
DR		10·9 (0·03)	0·44 (0·33)	1·23 (0·68)	2·39 (0·31)	0·16 (0·02)	14·6	
IRGC 117265								
BD1	Eqn (2), *K*_d_ and β_1_ constrained within BD treatments	10·5 (0·05)	0·57 (0·50)	0·48 (0·19)	3·40 (0·99)	0·09 (0·05)	36·3	108·6
BD2		10·5 (0·05)						
BD3		10·5 (0·05)						
BD4		10·5 (0·06)						
BD5		10·5 (0·06)						
BD6		10·5 (0·57)						
DR		10·6 (0·03)	0·88 (0·24)	0·45 (0·09)	2·63 (0·48)	0·15 (0·03)	17·4	
IRGC 117266								
BD1	Eqn (2), *K*_d_ and β_1_ constrained within BD treatments	10·9 (0·02)	1·25 (0·50)	0·05 (0·16)	7·71 (1·60)	0·26 (0·08)	30·2	66·9
BD2		10·9 (0·02)						
BD3		10·9 (0·03)						
BD4		10·9 (0·02)						
BD5		10·9 (0·02)						
BD6		10·8 (0·04)						
DR		10·7 (0·02)	0·89 (0·25)	0·15 (0·08)	4·00 (0·72)	0·22 (0·04)	18·1	
IRGC 117267								
BD1	Eqn (2), no parameter constrained	10·9 (0·06)	0·52 (0·44)	0·17 (0·05)	4·23 (2·54)	0·09 (0·07)	47·7	30·8
BD2^*^		10·9 (0·03)	–	–	–	–	–	
BD3		10·8 (0·03)	0·49 (0·43)	0·12 (0·05)	3·56 (2·56)	0·06 (0·06)	63·7	
BD4		10·8 (0·03)	0·80 (0·46)	0·22 (0·07)	3·52 (2·48)	0·07 (0·06)	54·2	
BD5		10·8 (0·03)	0·86 (0·46)	0·22 (0·07)	2·96 (2·45)	0·05 (0·06)	57·5	
BD6		10·7 (0·03)	0·60 (0·45)	0·18 (0·06)	3·37 (2·47)	0·06 (0·06)	56·3	
DR		10·7 (0·02)	0·32 (0·17)	0·07 (0·02)	5·46 (1·18)	0·11 (0·02)	48·7	
IRGC 117268								
BD1	Eqn (1), *K*_i_ and σ^–1^ constrained within all treatments (BD and DR)	10·7 (0·04)	–	–	2·78 (0·06)	0·08 (0·00)	37·2	0
BD2		10·7 (0·04)	–	–				
BD3		10·7 (0·03)	–	–				
BD4		10·6 (0·04)	–	–				
BD5		10·7 (0·03)	–	–				
BD6		10·7 (0·04)	–	–				
DR		10·7 (0·02)	–	–				
IRGC 117269								
BD1	Eqn (1), *K*_i_ and σ^–1^ constrained within BD treatments	11·5 (0·05)	–	–	2·69 (0·06)	0·19 (0·00)	14·3	155·4
BD2		11·5 (0·03)	–	–				
BD3		11·5 (0·11)	–	–				
BD4		11·7 (0·04)	–	–				
DR		11·6 (0·03)	–	–	1·13 (0·05)	0·20 (0·00)	5·6	
IRGC 117270								
BD1	Eqn (1), *K*_i_ and σ^–1^ constrained within BD treatments	11·0 (0·05)	–	–	3·28 (0·08)	0·19 (0·05)	17·2	149·3
BD2		10·8 (0·05)	–	–				
BD3		10·8 (0·03)	–	–				
BD4		10·8 (0·06)	–	–				
BD5		10·7 (0·07)	–	–				
BD6		10·6 (0·11)	–	–				
DR		10·9 (0·12)	–	–	1·39 (0·06)	0·20 (0·01)	6·9	
IRGC 117271								
BD1	Eqn (1), *K*_i_ and σ^–1^ constrained within all treatments (BD and DR)	10·6 (0·02)	–	–	3·89 (0·09)	0·13 (0·00)	29·0	0
BD2		10·6 (0·05)	–	–				
BD3		10·6 (0·02)	–	–				
BD4		10·6 (0·02)	–	–				
BD5		10·6 (0·02)	–	–				
BD6		10·6 (0·04)	–	–				
DR		10·6 (0·02)	–	–				
IRGC 117272								
BD1	Eqn (1), *K*_i_ and σ^–1^ constrained within all treatments (BD and DR)	11·1 (0·05)	–	–	3·60 (0·08)	0·09 (0·00)	41·0	0
BD2		11·1 (0·05)	–	–				
BD3		11·0 (0·05)	–	–				
DR		10·9 (0·05)	–	–				
IRGC 117273								
BD1	Eqn (2), no parameters constrained	10·6 (0·06)	0·18 (0·45)	0·32 (0·14)	4·22 (1·92)	0·23 (0·11)	18·4	70·5
BD2		10·8 (0·03)	0·03 (0·45)	0·20 (0·14)	5·85 (2·25)	0·32 (0·13)	18·2	
BD3		10·5 (0·24)	0·34 (0·44)	0·24 (0·12)	4·97 (2·11)	0·27 (0·12)	18·7	
BD4		10·8 (0·03)	0·12 (0·44)	0·19 (0·13)	4·55 (1·89)	0·20 (0·11)	23·1	
BD5		10·6 (0·03)	0·52 (0·44)	0·25 (0·12)	7·15 (2·22)	0·31 (0·12)	23·5	
BD6		10·3 (0·08)	0·26 (0·44)	0·13 (0·13)	4·37 (1·87)	0·16 (0·10)	28·3	
DR		10·8 (0·02)	0·08 (0·18)	0·19 (0·05)	4·77 (0·86)	0·29 (0·05)	16·6	
IRGC 117274								
BD1	Eqn (1), *K*_i_ and σ^–1^ constrained within BD treatments	10·7 (0·06)	–	–	0·94 (0·02)	0·11 (0·00)	8·5	372·2
BD2		10·8 (0·04)	–	–				
BD3		10·8 (0·04)	–	–				
BD4		10·8 (0·04)	–	–				
BD5		10·7 (0·03)	–	–				
BD6		10·8 (0·04)	–	–				
DR		10·8 (0·10)	–	–	0·26 (0·05)	0·14 (0·00)	1·8	
IRGC 117275								
BD1	Eqn (2), *K*_d_, β_1,_ *K*_i_ and σ^–1^ constrained within all treatments (BD and DR)	11·3 (0·06)	0·77 (0·09)	0·19 (0·03)	3·85 (0·18)	0·10 (0·01)	38·7	0
BD2		11·4 (0·16)						
BD3		11·3 (0·02)						
BD4		11·3 (0·04)						
BD5		11·2 (0·03)						
BD6		11·2 (0·07)						
DR		11·5 (0·25)						
IRGC 117276								
BD1	Eqn (1), σ^–1^ constrained within all treatments (BD and DR)	10·8 (0·02)	–	–	3·20 (0·10)	0·16 (0·00)	19·8	1·3
BD2		10·9 (0·02)	–	–	3·21 (0·10)		19·7	
BD3		10·9 (0·02)	–	–	3·89 (0·11)		24·1	
BD4		10·9 (0·02)	–	–	3·70 (0·11)		22·9	
BD5		10·9 (0·05)	–	–	3·87 (0·11)		24·0	
BD6		10·9 (0·02)	–	–	3·67 (0·11)		22·7	
DR		10·9 (0·05)	–	–	3·84 (0·11)		23·8	
IRGC 117277								
BD1	Eqn (1), *K*_i_ and σ^–1^ constrained within all treatments (BD and DR)	10·8 (0·03)	–	–	2·93 (0·05)	0·12 (0·00)	24·2	0
BD2		10·7 (0·02)	–	–				
BD3		10·7 (0·04)	–	–				
BD4		10·7 (0·03)	–	–				
BD5		10·5 (0·03)	–	–				
BD6		10·6 (0·03)	–	–				
DR		10·7 (0·03)	–	–				
IRGC 117278								
BD1	Eqn (1), σ^–1^ constrained within all treatments (BD and DR)	10·5 (0·05)	–	–	2·87 (0·06)	0·08 (0·00)	34·8	35·3
BD2		10·5 (0·04)	–	–	3·19 (0·06)		38·7	
BD3		10·5 (0·06)	–	–	3·09 (0·06)		37·4	
BD4		10·6 (0·05)	–	–	2·97 (0·06)		35·9	
BD5		10·6 (0·05)	–	–	2·70 (0·06)		32·7	
BD6		10·5 (0·02)	–	–	2·66 (0·06)		32·2	
DR		10·6 (0·04)	–	–	2·90 (0·10)		28·6	
IRGC 117279								
BD1	Eqn (1), *K*_i_ and σ^–1^ constrained within BD treatments	11·0 (0·06)	–	–	3·99 (0·08)	0·17 (0·00)	24·1	23·6
BD2		11·0 (0·04)	–	–				
BD3		11·1 (0·03)	–	–				
BD4		11·1 (0·03)	–	–				
BD5		11·1 (0·03)	–	–				
BD6		11·2 (0·06)	–	–				
DR		11·0 (0·07)	–	–	3·23 (0·08)	0·17 (0·00)	19·5	
IRGC 117280								
BD1	Eqn (1), σ^–1^ constrained within all treatments (BD and DR)	11·3 (0·20)	–	–	2·96 (0·09)	0·16 (0·00)	18·8	95·1
BD2		11·2 (0·03)	–	–	3·82 (0·11)		24·2	
BD3		11·0 (0·04)	–	–	3·29 (0·10)		20·9	
BD4		11·0 (0·02)	–	–	3·89 (0·11)		24·7	
BD5		11·0 (0·02)	–	–	3·68 (0·11)		23·4	
BD6		11·0 (0·03)	–	–	4·40 (0·12)		27·9	
DR		11·1 (0·02)	–	–	2·25 (0·07)		14·3	
IRGC 117281								
BD1	Eqn (2), no parameters constrained	10·9 (0·03)	1·05 (0·61)	0·45 (0·17)	3·05 (1·38)	0·09 (0·05)	33·1	50·4
BD2		10·9 (0·04)	0·42 (0·54)	0·11 (0·13)	5·80 (1·81)	0·17 (0·06)	34·2	
BD3		10·9 (0·03)	0·17 (0·56)	0·18 (0·14)	4·19 (1·53)	0·14 (0·05)	29·8	
BD4		10·9 (0·05)	0·52 (0·52)	0·05 (0·12)	9·61 (3·06)	0·23 (0·08)	42·4	
BD5		10·6 (0·02)	0·03 (0·57)	0·27 (0·16)	5·65 (1·69)	0·16 (0·05)	34·4	
BD6		10·7 (0·04)	0·61 (0·54)	0·08 (0·13)	6·52 (2·06)	0·17 (0·06)	39·6	
DR		10·9 (0·03)	0·27 (0·23)	0·23 (0·06)	4·13 (0·63)	0·15 (0·02)	28·2	
IRGC 117282								
BD1	Eqn (1), *K*_i_ and σ^–1^ constrained within BD treatments	10·9 (0·06)	–	–	2·83 (0·06)	0·22 (0·00)	12·8	21·9
BD2		11·0 (0·04)	–	–				
BD3		10·9 (0·03)	–	–				
BD4		10·8 (0·04)	–	–				
BD5		10·8 (0·06)	–	–				
BD6		10·8 (0·07)	–	–				
DR		11·0 (0·06)	–	–	2·35 (0·00)	0·22 (0·00)	10·5	
IRGC 117283								
BD1	Eqn (2), *K*_d_, β_1,_ *K*_i_ and σ^–1^ constrained within all treatments (BD and DR)	10·9 (0·05)	0·05 (0·05)	0·10 (0·01)	3·47 (0·19)	0·11 (0·01)	32·8	0
BD2		11·0 (0·06)						
BD3		11·0 (0·06)						
BD4		10·8 (0·05)						
BD5		10·7 (0·06)						
DR		10·8 (0·06)						

^*^Unable to fit probit model.
