# Supplementary material for: Increases in the longevity of desiccation-phase developing rice seeds: response to high-temperature drying depends on harvest moisture content
Source: Ann Bot. 2015 Jul 1;116(2):247–59. doi: 10.1093/aob/mcv091 (PMC4512194; doi:10.1093/aob/mcv091)
Supplement: Supplementary Data [file supp_116_2_247__index.html]

Increases in the longevity of desiccation-phase developing rice seeds: response to high-temperature drying depends on harvest moisture content — Supplementary Data 

# Increases in the longevity of desiccation-phase developing rice seeds: response to high-temperature drying depends on harvest moisture content

## Supplementary Data

files

- Supplementary Data - pdf file
