# Supplementary material for: Increases in the longevity of desiccation-phase developing rice seeds: response to high-temperature drying depends on harvest moisture content
Source: Ann Bot. 2015 Jul 1;116(2):247–59. doi: 10.1093/aob/mcv091 (PMC4512194; doi:10.1093/aob/mcv091)
Supplement: Supplementary Data [file supp_mcv091_aob-15239-s01.pdf]

SUPPLEMENTARY DATA

Fig. S1. Ability to germinate during storage at 45°C and a m.c. of 10.9% for seeds of 20 rice accessions dried immediately in the dryroom (DR) or initially dried in the batch dryer for 1 (BD1), 2 (BD2), 3 (BD3), 4 (BD4), 5 (BD5) or 6 (BD6) days. Eqn (1) or Eqn (2) were fitted to the data with or without parameter constraints; the results shown are for the model with the fewest parameters that could be fitted without a significant increase in residual deviance compared with the best-fit model (see Appendix in main text).

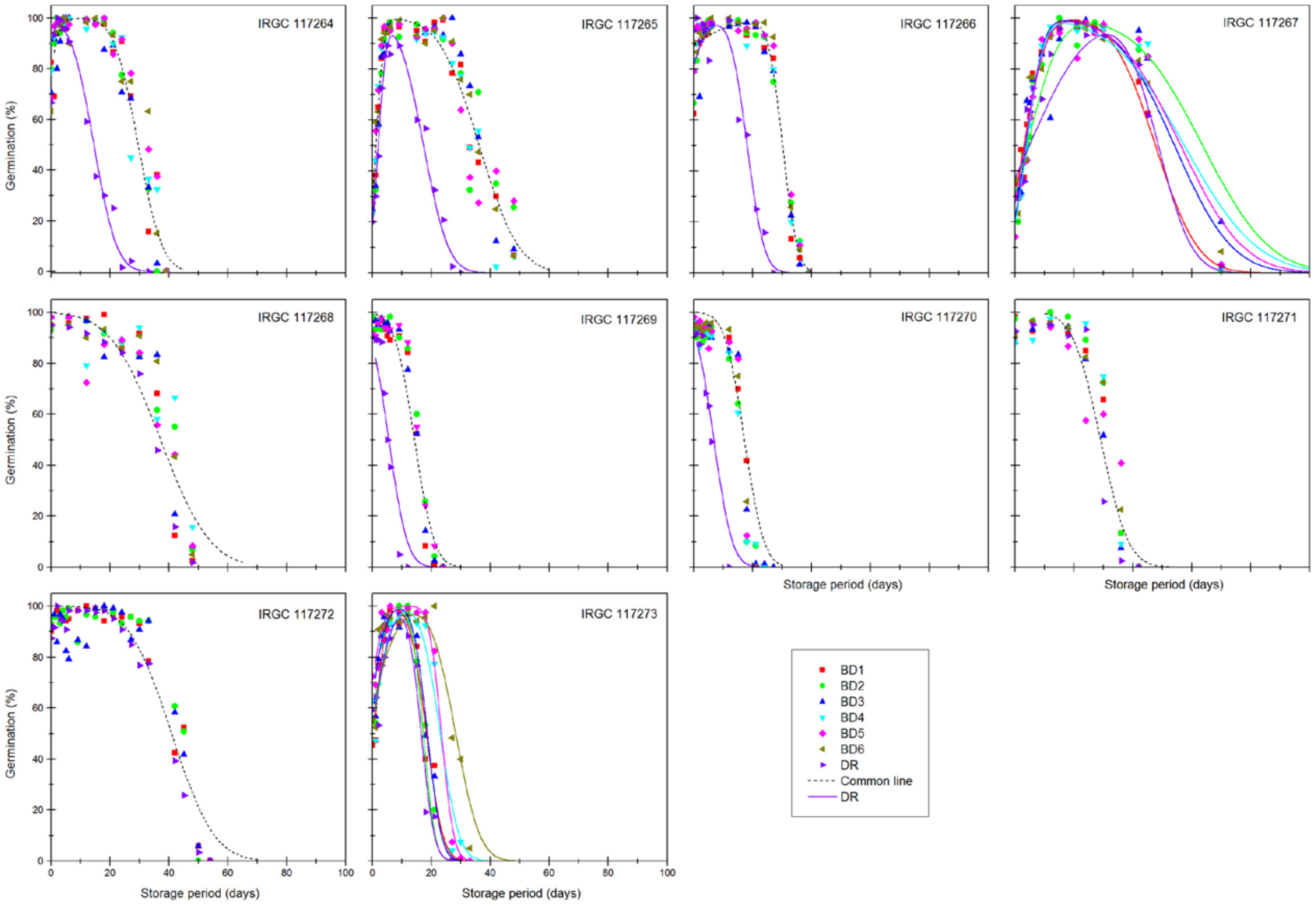

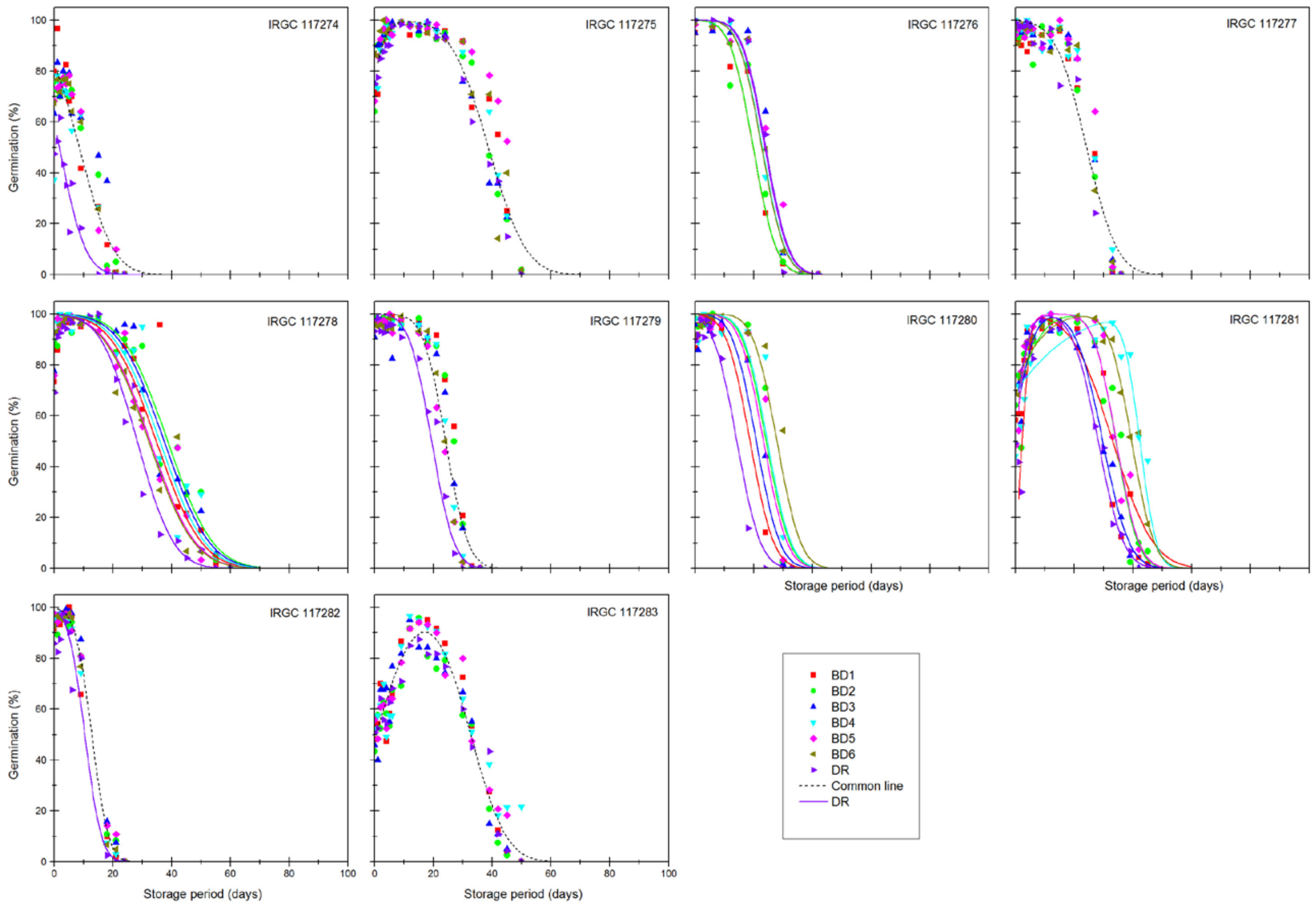

IRGC 117274
IRGC 117275
IRGC 117276
IRGC 117277
IRGC 117278
IRGC 117279
IRGC 117280
IRGC 117281
IRGC 117282
IRGC 117283
Germination (%)
Storage period (days)
BD1
BD2
BD3
BD4
BD5
BD6
DR
Common line
DR
